# Research on weed identification in soybean fields based on the lightweight segmentation model DCSAnet

**DOI:** 10.3389/fpls.2023.1268218

**Published:** 2023-12-05

**Authors:** Helong Yu, Minghang Che, Han Yu, Yuntao Ma

**Affiliations:** ^1^ College of Information Technology, Jilin Agricultural University, Changchun, China; ^2^ College of Land Science and Technology, China Agricultural University, Beijing, China

**Keywords:** lightweight semantic segmentation, weed recognition, deep learning, encoder-decoder, convolutional neural network

## Abstract

Weeds can compete with crops for sunlight, water, space and various nutrients, which can affect the growth of crops.In recent years, people have started to use self-driving agricultural equipment, robots, etc. for weeding work and use of drones for weed identification and spraying of weeds with herbicides, and the effectiveness of these mobile weeding devices is largely limited by the superiority of weed detection capability. To improve the weed detection capability of mobile weed control devices, this paper proposes a lightweight weed segmentation network model DCSAnet that can be better applied to mobile weed control devices. The whole network model uses an encoder-decoder structure and the DCA module as the main feature extraction module. The main body of the DCA module is based on the reverse residual structure of MobileNetV3, effectively combines asymmetric convolution and depthwise separable convolution, and uses a channel shuffle strategy to increase the randomness of feature extraction. In the decoding stage, feature fusion utilizes the high-dimensional feature map to guide the aggregation of low-dimensional feature maps to reduce feature loss during fusion and increase the accuracy of the model. To validate the performance of this network model on the weed segmentation task, we collected a soybean field weed dataset containing a large number of weeds and crops and used this dataset to conduct an experimental study of DCSAnet. The results showed that our proposed DCSAnet achieves an MIoU of 85.95% with a model parameter number of 0.57 M and the highest segmentation accuracy in comparison with other lightweight networks, which demonstrates the effectiveness of the model for the weed segmentation task.

## 1 Introduction

Soybean belongs to the legume family, its production is second only to rice, wheat and corn, and it is one of the most important cash crops in the world (Gebregziabher et al., 2022; Huang et al., 2022). Soybean contains approximately 40% protein and 20% oil and is an important source of nutritious food for humans and a major source of feed for livestock. The healthy growth of the soybean plant plays a very important role in the yield of soybeans(Saito et al., 2021). Soybean plants are relatively small and susceptible to weeds (Soltani et al., 2022), Some studies have shown that the number of seeds contained in seed pods in a soybean field is negatively correlated with weed density (Naseri and Nazer Kakhki, 2022). Therefore, weed control is a key task in soybean cultivation, as yield and quality are affected by pests, diseases, and weeds throughout the growth cycle of soybean, with global soybean production losses approaching 30% annually(Khan et al., 2021).

Weed control using herbicides is currently the most common form of weed control (Zou et al., 2021b) and is widely used in soybean field weeding work, if herbicides are used at the right stage of soybean growth, they can suppress the weed population and thus increase the 100-grain weight of soybeans (Kakhki et al., 2022). However, the excessive use of chemical herbicides not only causes herbicides to be wasted but also leads to environmental contamination, including soil contamination and groundwater contamination (Dai et al., 2019). To reduce the misuse of chemical herbicides, we use precision agriculture, which can be defined as the technology applied to improve the efficiency of pesticide use and to protect the environment by implementing accurate management and distributing the exact dose of pesticide input in the right place. Weed detection and location technology is essential if we want to achieve this goal (Bah et al., 2019). If accurate detection of weeds can be achieved, not only can herbicide abuse be reduced, but appropriate herbicides can also be selected for different types of weeds. Some studies have used deep learning algorithms to detect weeds and display the results by drawing bounding boxes or pixel-level classification to develop a weed recognition system with good experimental results in effectively identifying the weeds (Tang et al., 2020; Wu et al., 2021). In soybean fields, there are mainly grass weeds, such as Matang and dogwood, and broadleaf weeds, such as ashwagandha, spicebush, iron amaranth, reverse amaranth, and concave-headed amaranth, which are very different from one another; if different herbicides are used for different families of weeds, better weed control results will be achieved (dos Santos Ferreira et al., 2017).

Many researchers have used traditional machine learning methods for weed identification work. Traditional machine learning-based algorithms use feature descriptors to extract object features from sensory data and use machine learning-based classifiers for classification, detection, or segmentation. Machine algorithms includes supervised learning algorithms, such as the k-nearest neighbor algorithm and logistic regression; and unsupervised learning algorithms, such as clustering and principal component analysis (PCA) (Kapach et al., 2012). Detection of weed work using machine learning algorithms (Random Forest (RF), Support Vector Machines (SVM) and k-nearest neighbor (KNN)) on drone images collected from chilli fields in Australia can yield high accuracy rates, with weed detection accuracies of 96%, 94%, and 63% for RF, SVM, and KNN, respectively (Islam et al., 2021), respectively. In weed detection in tobacco fields,an SVM classifier based on texture, shape and color with classification accuracy up to 96% and achieved a detection speed of 6 FPS (Tufail et al., 2021). However, traditional machine learning methods are unable to extract features autonomously, but require manually designed extracted features (including color information, location information, texture information, etc.), which limits the popularization and application of traditional machine learning in the field of weed recognition (Espejo-Garcia et al., 2020).

In recent years, deep learning-based detection methods have become the dominant approach in the field of weed identification(Espejo-Garcia et al., 2020; Liu and Bruch, 2020; Wu et al., 2021; Subeesh et al., 2022; Weng et al., 2022; (Peng et al., 2022). Deep learning does not require the human setting of what features are available and how to extract them. It automatically learns from the provided data to obtain the desired features (Fuentes-Pacheco et al., 2019), which can be used in a wide range of applications in agriculture. In weed recognition work, there are problems such as different weed sizes and shapes, weeds and crops obscuring one another, and too-dense recognition targets. To better cope with these problems, many studies have used semantic segmentation algorithms in deep learning for weed recognition. A semantic segmentation algorithm based on deep learning can achieve the segmentation of objects with irregular contours and densely distributed objects due to its feature of classifying objects pixel by pixel to identify weeds (Quan et al., 2021). Currently, many semantic segmentation models have been applied to weed recognition tasks; some commonly used ones include SegNet (Badrinarayanan et al., 2017), U-Net (Ronneberger et al., 2015), and DeepLab (Chen et al., 2014; Chen et al., 2017; Sandler et al., 2018) series models. K Zou et al. (Zou et al., 2021a) proposed a simplified U-Net model using weights pretrained on a classical dataset and fine-tuning the training method in two stages, which achieved a cross-merge ratio (IoU) of 92.91%. Z Wu et al. (Wu et al., 2021) conducted segmentation experiments on the degree of wilting of abnormal leaves of hydroponic lettuce using multiple DeepLabV3+ networks that used different feature extraction backbones. Comparing the results, they showed that the highest accuracy of the results was achieved when using ResNet101 as the backbone with an mIoU of 0.8326%, and the fastest recognition speed was achieved when using ResNet50 as the backbone. The recognition speed was only 154.0 ms per image. All of the above algorithmic models have good performance in terms of accuracy; however, they usually have high computational cost and long inference time due to their large number of network parameters or the need for large floating-point operations per second, or both. Currently, mobile and embedded devices are widely used with limited storage space and processor performance; thus, the number of parameters and computations prevent further application of these network models to mobile end devices (Tang et al., 2020; Lan et al., 2021; Weng et al., 2022).

To overcome the abovementioned drawbacks and to better apply detection algorithm models to mobile devices, many lightweight segmentation algorithms have been proposed in recent years and applied in the field of agriculture. Lan et al. (Lan et al., 2021) added two lightweight feature extraction backbones, MobileNetV2 (Sandler et al., 2018) and BiSeNetV2 (Yu et al., 2021), to the U-Net model and showed that the network parameters, model size, and computational effort of both were substantially reduced, with the three metrics of MobileNetV2 U-Net being reduced by 89.12%, 86.16% and 92.6%, the inference speed being increased by 2.77 times, and the recognition accuracy being 78.77%, which meets the accuracy and parametric size requirements for mobile networks. Zhou et al. (Zhou et al., 2020) developed an Android application called KiwiDetector for field kiwi detection using a single shot multibox detector (SSD) with two lightweight backbones, MobileNetV2 and InceptionV3 (Szegedy et al., 2016), with model sizes of 17.5 M and 24.1 M, respectively, and recognition accuracies of 90.8% and 72.8%, respectively. The results show that deep learning algorithms using lightweight networks can be embedded easily in mobile devices and can achieve high detection accuracy.

In this study, to further investigate the lightweight problem of the soybean field weed recognition model, reduce the memory requirement of mobile devices such as UAVs and improve the detection accuracy, an improved lightweight model DCSAnet is proposed and applied to the soybean field weed recognition task. We conducted an experimental study of segmentation using DCSAnet and other classical segmentation models on a self-acquired soybean field image dataset. The goal of this experiment is to classify the target pixels into soybean, graminoid weeds, broadleaf weeds and background, and select the most suitable lightweight segmentation model by comparing the segmentation results.

## 2 Materials and methods

### 2.1 Data acquisition and preprocessing

#### 2.1.1 Image acquisition

The soybean weed dataset used in this experiment was collected from a soybean ex-perimental field at Jilin Agricultural University in Changchun, Jilin Province, China, be-tween 9:00 and 15:00 on June 10 and 16,2021. The device used was a Huawei mate30 cell phone, with a shoot-ing angle perpendicular to the ground, a distance of 60 cm from the ground, a resolution of 3000×4000 pixels, and JPG format images. A total of 119 larger original images were acquired, the data image mainly contains soybean crops, graminoid weeds such as Digitaria san-guinalis (L.) Scop and Setaria viridis (L.) Beauv and broadleaf weeds such as Chenopodi-um glaucum L,Acalypha australis L,and Amaranthus retroflexus L, as well as back-ground consisting of soil, stones, and dead plants. The distribution of weeds and crops in the dataset used in this experiment is complex, containing a large number of weeds and crops shading each other, which makes identification difficult.

#### 2.1.2 Image preprocessing

**Figure 1:**
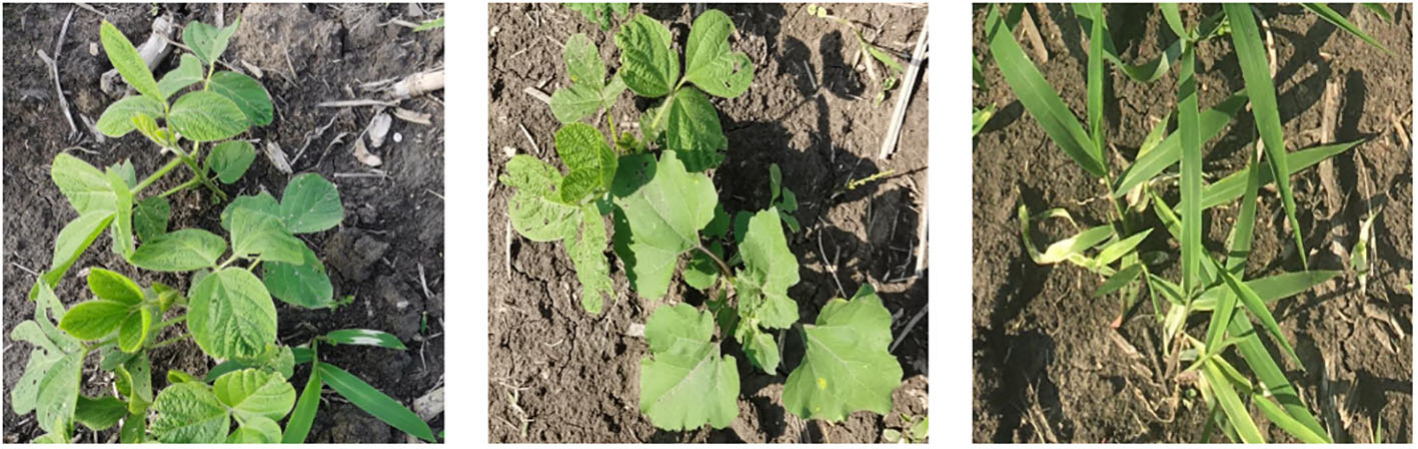
Examples of images of selected datasets.

The length and width of the image is higher than 2000 pixels, because of the images are large, direct recognition would increase the burden on the network model; therefore, the first 520 images of 512×512 pixels were obtained by random cropping, then, some unclear images were eliminated, resulting in 482 images. Examples of some soybean field weed images are shown in **Figure 1**.

**Figure 2:**
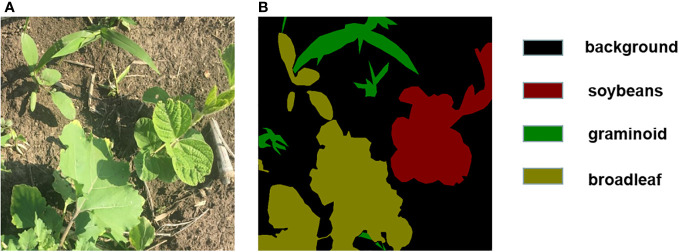
**(A)** The original image; **(B)** labeled image.

We used the labeling tool LabelMe to label different categories of pixels in the image (Russell et al., 2008), and classified the image pixels into four categories including soybean, graminoid weeds, broadleaf weed, and background,Labeling results are shown in **Figure 2**. To enhance the robustness and generalization of the model, we expanded the dataset by using random rotation, flipping, adding Gaussian noise, and increasing contrast. The expanded dataset has 2410 images, which are randomly divided into training, validation, and test sets in the ratio of 6:3:1.

### 2.2 Model structure

**Figure 3:**
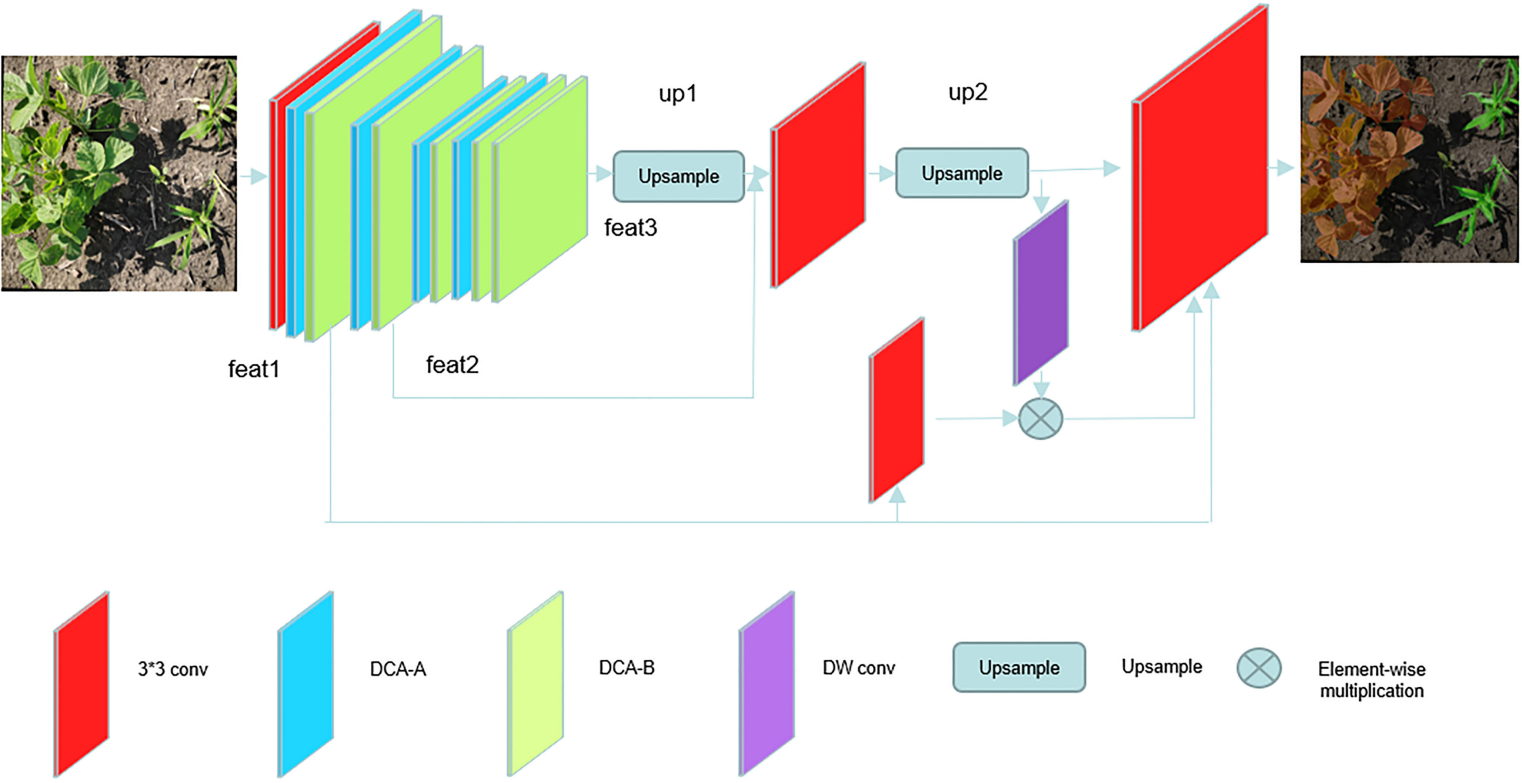
DCSAnet model structure diagram.

Our proposed model DCSAnet aims to improve the integrated capability of weed identification for mobile devices in agricultural production,such as UAVs and unmanned weeding equipment. This requires a trade-off between detection accuracy and the number of model parameters; the model structure is shown in **Figure 3**. To reduce the number of model parameters and improve the detection speed, we design a backbone network with only 12 layers for feature extraction in the coding layer and generate three feature maps of different sizes. The coding stage consists of a 3×3 convolution with a step size of 2 and three stages. The 3×3 convolution can initially extract the features and reduce the feature map from the original size to reduce the number of parameters in process of feature extraction, and then the subsequent three stages complete the feature extraction work. The first two stagesboth consisting of a DCA-B and a DCA-A, which can realize downsampling and preliminary feature extraction, and these two stages generate shallow feature maps feat1 and feat2 with more spatial information.

The main role of the third stage is to obtain deep feature maps containing a large amount of semantic information. To achieve this goal, we use more feature extraction modules in this stage and use the channel attention mechanism to enhance the weights of important feature channels and finally generate a feature map feat3 with size 1/32 of the original map and containing more semantic information, our proposed model improves the utilization of spatial information without increasing the number of parameters.

In the decoding stage, to better fuse feature maps at different scales, we borrowed from U-Net’s decoding approach by using deep feature maps for upsampling in steps and fusing feature maps at different scales in the process to compensate for the loss of spatial information in the encoding process (Ronneberger et al., 2015; Cao et al., 2020). However, directly stitches the two feature maps along the channel dimension ignores the correlation of relevant location information between different feature maps, which will lead to the lack of utilization of information between different layers. To better use different dimensional feature maps (Yu et al., 2021), we borrowed the idea of guided aggregation to use the high-dimensional feature map containing more semantic information to guide the feature construction of the low-dimensional feature map in the fusion operation of different dimensional feature maps. The specific implementation is to first extract the features of the high-dimensional feature map using a 3×3 convolution with 2 steps and a batch normalization operation, fuse them with the upper layer feature map by an elementwise multiplication operation, and then use the fused feature map to join the fusion operation of feature maps of different dimensions. Our Method achieve effective communication between feature maps, reduced missing information in the feature map fusion process.

#### 2.2.1 Depthwise channel shuffle asymmetric module

**Figure 4:**
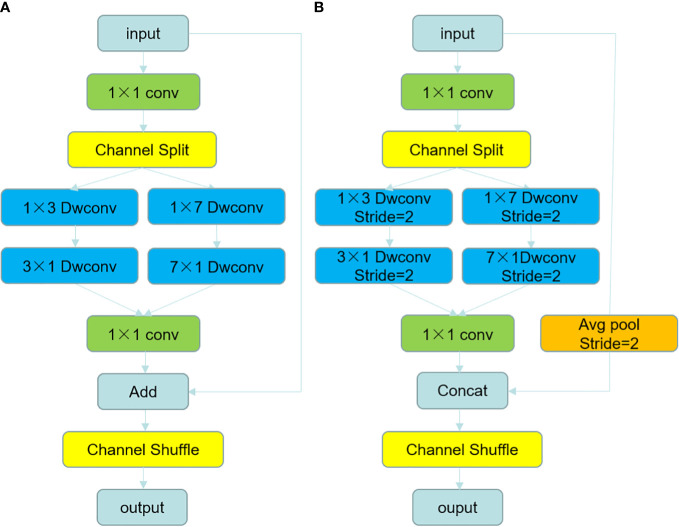
The structure diagram of DCA-A module and DCA-B module, **(A)** shows the structure of DCA-A module and **(B)** shows the structure of DCA-B module.

Inspired by the linear bottlenecks in MobileNetV3 and the channel shuffle in ShuffleNet, we combined the advantages of both and designed the feature extraction modules DCA-A and DCA-B. The structure of both modules is shown in **Figure 4**. Between them, DCA-A is used for in-stage feature extraction, and DCA-B is used for feature extraction and to implement the downsampling function. The feature extraction of the modules mimics the inverted residuals structure in MobileNetV3. The specific process is to first use a 1×1 convolution to extend the dimensionality in the head of each module and extend the number of channels of the feature map to 4 times the input because the higher the number of channels, the better the feature extraction will be.

Using convolution kernels with different scales can extract more multiscale features, but it will increase the amount of computation. To extract multiscale features without increasing the computation, we next perform channel splitting of the feature maps, splitting them into two feature maps x1 and x2 with half the number of channels in channel order, and use asymmetric depth-separable convolution with different convolution kernel sizes for feature extraction. Two convolution kernels with different feeling fields can extract features of different scales, and the channel of each branch has only half of the original feature dimension, so there is no increase in computational effort.

Ordinary 2D convolution uses convolution kernels of equal length and width and computes both the channel and space of the feature map at the same time, which increases the number of parameters of the model and the size of the occupied memory. To achieve a lightweight model, we use a depth-separable strategy and 1D convolution kernels. The specific process is to use asymmetric depth-separable convolution kernels of size N × 1 and 1 × N in two channels consecutively. Instead of the 2D depth-separable convolution with N×N convolution kernels, feature extraction is performed to further reduce the number of parameters. After feature extraction, the two feature maps extracting different perceptual field information are then stitched together and downscaled by a 1×1 convolution for the purpose of compressing and integrating dimensional features and accelerating the overall computation speed.

In the feature extraction module DCA-A, we perform a channel-by-channel elementwise addition of the input feature map and the reduced-dimensional feature map to achieve residual connectivity, which can serve the purpose of preventing network degradation (Zhuang et al., 2021). To prevent the problem that different branches can only train on a fixed half of the channels due to channel splitting, we use a channel shuffling operation to disrupt the order of the channels after residual connection to achieve information interaction between different branches and realize a complete feature extraction process.

In the DCA-B module, we use an asymmetric depth-separable convolution with a step stride of 2 to halve the size of the resulting feature map after feature extraction; make maximum pooling to halve the size of the input feature map before residual concatenation; and then use Concat to stitch the input feature map with the resultant feature map by channel direction to halve the size of the feature map and double the number of channels. Then, the channel shuffling operation disrupts the order of channels to achieve downsampling.

#### 2.2.2 Depthwise separable convolution and asymmetric convolution

The convolution operation can automatically extract features from the input feature map through the weight learning function. The traditional convolution has a convolution kernel of equal length and width and the same depth as the input feature map. The computational process is to use *C*_2_ convolution kernels of size $\left(N\times N\times C_{1}\right)$ to slide through the input feature map $\left(H\times W\times C_{1}\right)$ along the height and width directions to generate an output feature map of size $\left(H\times W\times C_{2}\right)$ . The computational volume of the standard convolution is as follows:


$$H\times W\times N\times N\times C_{1}\times C_{2}$$

In the deep separable convolution, the convolution operation is divided into deep and pointwise convolutions of (1 × 1) size (Howard et al., 2017). Deep convolution is performed using *C*_1_ 2D convolution kernels of size (N×N×1) , each of which operates on one channel of the input feature map separately. The final results are stacked together to generate a feature map of size $\left(H\times W\times C_{1}\right)$ . Then, point-by-point convolution is performed by *C*_2_ $\left(1\times 1\times C_{1}\right)$ convolution kernels to generate an output feature map of size $\left(H\times W\times C_{2}\right)$ . The computational volume of the depth-separable convolution is as follows:


$$H\times W\times N\times N\times C_{1}+H\times W\times C_{1}\times C_{2}$$

The computational effort of the deep separable convolution compared to the normal convolution is as follows:


$$\frac{H\times W\times N\times N\times C_{1}+H\times W\times C_{1}\times C_{2}}{H\times W\times N\times N\times C_{1}\times C_{2}}=\frac{1}{C_{2}}+\frac{1}{N^{2}}$$

Asymmetric convolution replaces the standard convolution by using two consecutive convolutions: (N × 1) and (1 × N) (Wang et al., 2019; Hu and Gong, 2021), which results in a significant reduction in computational effort by sacrificing a certain amount of accuracy. The computational effort of asymmetric convolution compared to ordinary convolution is as follows:


$$\frac{H\times W\times N\times 1\times C_{1}\times C_{2}+H\times W\times 1\times N\times C_{1}\times C_{2}}{H\times W\times N\times N\times C_{1}\times C_{2}}=\frac{2}{N}$$

#### 2.2.3 Channel shuffling

**Figure 5:**
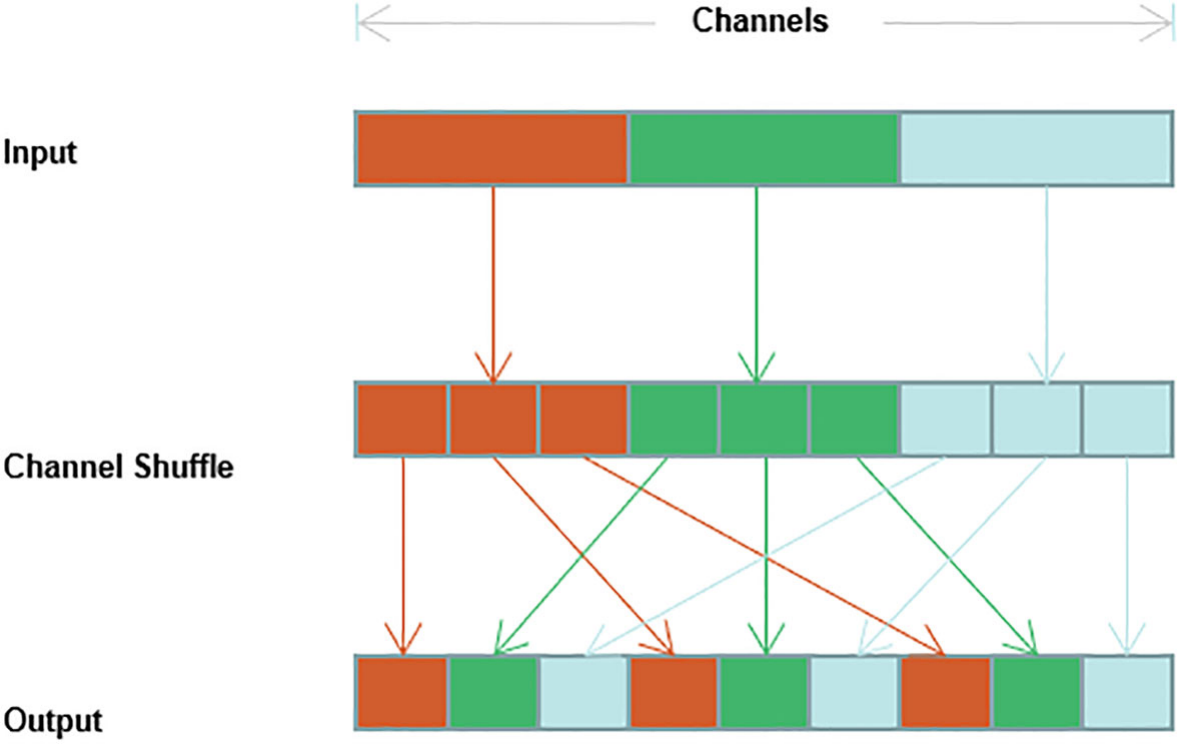
Implementation process of channel shuffling operation.

To reduce the number of parameters for the convolution operation, many model algorithms group the input feature maps by channel such that smaller convolution kernels can be used for sparse computation to reduce the number of operations; however, grouped convolution can lead to the inability of the channels in different groups to interact with features, which can lead to a weakened feature extraction capability of the convolution operation. If the channel random mixing operation is added after the grouped convolution, the original channel order can be disrupted such that the channels contained in the group are different for each grouping to achieve channel feature interactions for the whole channel (Ma et al., 2018; Zhang et al., 2018; Zhuang et al., 2021). The implementation process of the channel shuffling operation is shown in **Figure 5**.

The specific operation of channel shuffling divides the input feature map into g groups in the order of channel dimensions, with n channels in each group. The total number of channels is N=g×n , and then the transpose operation is used to become (n×g) . The result is then spread back to N dimensionsto disrupt the channels and avoid the situation that the group convolution cannot learn the full channel features.

### 2.3 Model training

The server environment used for this experiment is Windows 10, Python version 3.8.13, PyTorch version 1.7.1, and CUDA version 11.3. The experiment is run on a GPU with an NVIDIA Quadro RTX 8000 dedicated graphics card and 48 GB of graphics memory.

In the dataset used in this experiment, the pixel share of soybean leaves is higher than the share of other species of weeds, which can lead to positive and negative sample imbalance problems. To better reduce the impact of category imbalance on the results, this experiment uses a cross-entropy loss function to measure the loss between classes, which is calculated as follows:


$$Cross\_entropy=-\frac{1}{N}\sum_{i}\sum_{c=1}^{M}y_{ic}\log(p_{ic})$$

where M is the number of categories; y_ic is the sign function, equal to 1 if the true category of sample i is equal to c and 0 otherwise; and p_ic is the predicted probability that the observed sample i belongs to category c.

When performing network training, the size of the learning rate will have an impact on the convergence speed and final accuracy of the model (Chai et al., 2020; Zhuang et al., 2021); a smaller learning rate will lead to slow convergence, while a larger learning rate will lead to difficult convergence of the final result. To adjust the learning rate adaptively, we used the Adam optimizer to adjust the learning rate so that the learning rate can follow the frequency change of the parameters. The Adam initial learning rate of the optimizer is 0.001, the batch size is 4, and the number of iterations of the model is 400.

### 2.4 Evaluation indicators

To achieve a lightweight soybean field weed segmentation model, the goal of this experiment is to balance the detection accuracy and model size to achieve a high detection accuracy with a small number of model parameters; therefore, the mean intersection-to-merge ratio (MIoU), the number of model parameters (Params), and the number of billion floating point operations per second (GFLOPS) are used to evaluate the model accuracy and size.


$$MIoU=\frac{1}{k+1}\sum_{i=0}^{k}\frac{TP}{TP+FN+FP}$$

where TP is pixel detection as positive and true label as positive, FN is pixel detection as negative but true label as positive, FP is pixel detection as positive but true label as negative, and TN is pixel detection as inverse and true label as inverse, k is the number of categories.

## 3 Results and discussion

### 3.1 Ablation experiments

In this section, we conduct ablation experiments on the encoding part and the decoding part of the DCSAnet model separately verifying the validity of each part of our model.

#### 3.1.1 Encoding section

Our encoding part mainly borrows ideas from MobileNetv3; therefore, we use the U-Net model with the backbone of MobileNetv3 as the original model and compare it with the model using different improvement points. To verify the effect of using different sizes of asymmetric convolution in our feature extraction module DCA-A on the experimental results, we compare the experimental results when the feature extraction module uses asymmetric convolution with convolution kernel sizes of 5×1, 7×1, and 9×1 and the results without the channel blending operation. Our results are shown in **Table 1**.

**Table 1:** Experimental results under different improvement points in Encoding stage.

Model	kernel size	Channel Shuffle	*FLOPs* (*G*)	*Param* (*M*)	*MIoU* (%)
MobileNet3-Unet	——	——	16.67	2.434	83.89
DCSA	5×1	√	16.97	0.530	84.63
DCSA	7×1	√	17.00	0.534	84.86
DCSA	7×1	——	17.00	0.454	84.71
DCSA	9×1	√	17.12	0.467	84.79

√ shows that the models in the horizontal columns contain Channel Shuffle structures.

MobileNet3-U-Net, used as a benchmark comparison, obtained 83.89% MIoU with a model size of 2.434 M. In contrast, our DCSA achieved 84.63%, 84.86% and 84.79% MIoU with asymmetric convolutional kernel sizes of 5×1, 7×1 and 9×1, respectively, and the number of parameters decreased considerably. Subsequently, we also compare the experimental results of the DCSA model with an asymmetric convolutional kernel size of 7 × 1 after removing the channel shuffle operation. The MIoU decreases by 0.15% after removing the channel shuffle, which shows that the optimal experimental results can be obtained using an asymmetric convolutional kernel of size 7 × 1 and channel shuffle.

#### 3.1.2 Decoding section

In the decoding section, we compare the effects of using different feature map fusion methods on the experimental results. We use the output feature map of an encoder with a convolutional kernel size of 7 × 1 as the input to the decoding operation and use different decoding strategies. The results are shown in **Table 2**.

**Table 2:** Experimental results under different improvement points in decoding stage.

Model	*FLOPs* (*G*)	*Param* (*M*)	*MIoU* (%)
DCSA-0	17.00	0.45	84.86
DCSA- feat2-1	18.26	0.60	85.28
DCSA- feat3-1	18.17	0.55	85.22
REDCSA- feat2-1	17.98	0.62	85.62
REDCSA- feat3-1	18.06	0.57	85.95

First, we used the strategy of gradually upsampling the highest-dimensional feature map in U-Net and gradually fusing other feature maps in the process; the results are shown in DCSA-0. At this point, the MIoU is 84.86%. Next, we compared the experimental results after using feat3 or feat2 to perform guided aggregation on feat1 before fusing with up2, to form DCSA- feat2-1 and DCSA- feat3-1, respectively. The results were improved by 0.42% and 0.36%, respectively, showing that using only a high-dimensional feature map to a low-dimensional feature map for guided aggregation has limited improvement on the experimental results. Next, we compared the experimental results of REDCSA- feat2-1 and REDCSA- feat3-1 by adding feat1 before performing guided aggregation as residuals to the fusion of feat1 with up2 after bootstrap aggregation, at which point the results were improved by 0.76% and 0.9%, respectively, where using feat3 to feat1 for guided aggregation and adding residuals can obtain the best results. For this result, we believe that although the guided aggregation operation can reduce the feature fusion effect in the fusion stage, it may also bring about the loss of feature information, while the best feature fusion effect can be achieved after using the residual connection.

### 3.2 Comparison of DCSAnet with other methods

To verify the effectiveness of DCSAnet on the soybean weed detection task, we compared it with several lightweight classical semantic segmentation models of similar parametric size, including the original U-Net model with VGG (Simonyan and Zisserman, 2014) as the backbone, the U-Net model with MobileNetV3 replaced by the backbone network, the backbone network with the recently proposed ViT transformer’s segmentation model MobileNetViT (Mehta and Rastegari, 2021), CGNet (Wu et al., 2020), and LEDNet (Wang et al., 2019). All models are consistent with the experimental environment and experimental parameters.

**Figure 6:**
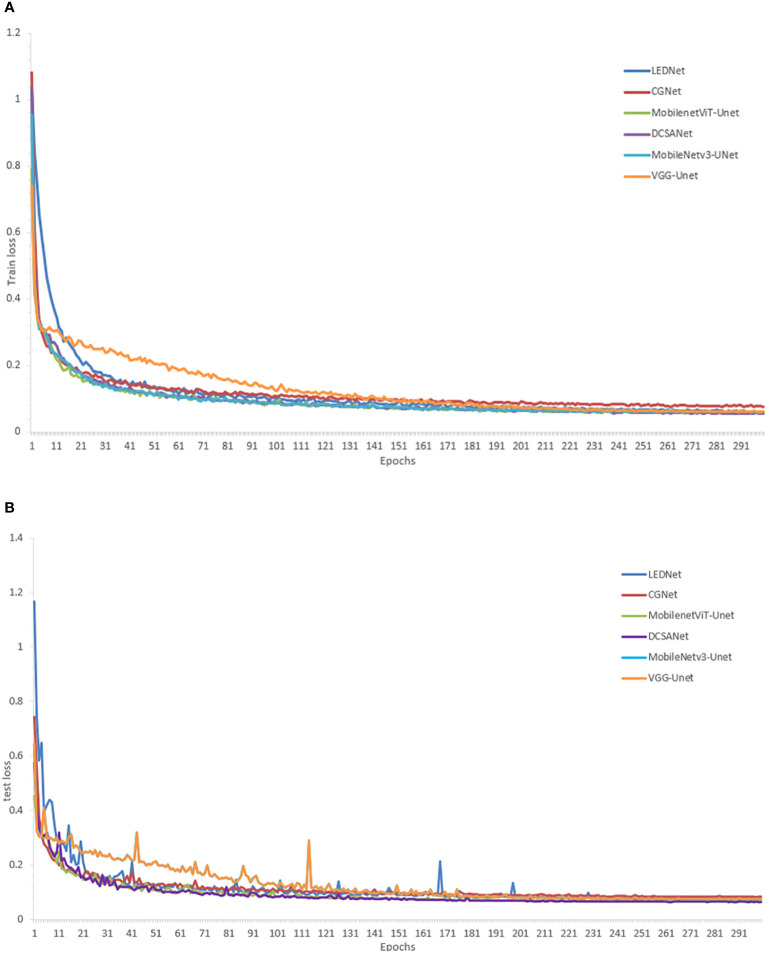
Loss variation plots **(A)** loss plots for the training set; **(B)** loss plots for the test set.

First, the loss curves of the different models are shown in **Figure 6**. The training losses in the figure show that the training results of all models eventually converge, which shows that all models can be used for field weed detection work. The test set losses in the figure show that MobileNet3-U-Net and DCSANet fluctuate the least in this process and both reach the smallest losses among these models, but DCSANet has a faster initial drop and converges faster, thus reaching the training requirements faster.

**Figure 7:**
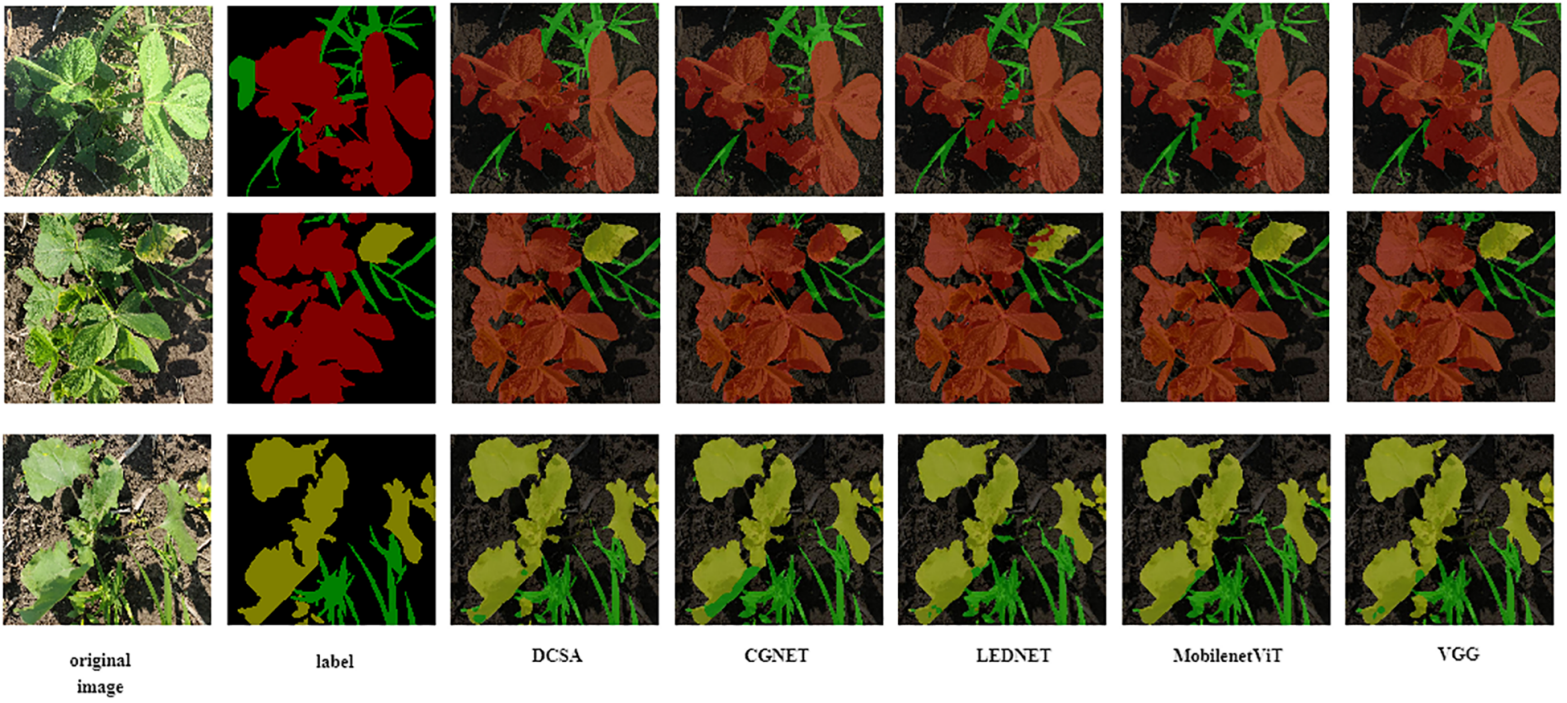
Segmentation results of different models on soybean field weed dataset Figure.

Then, we analyzed the accuracy and model size of different models. As shown in **Table 3**, the FLOPS of our model is 18.06 G, and the MIoU is 85.95%; that is, we improved the MIoU by 2.06% with only a 1.59 G increase in the gigabit floating point per second of the model and kept the size of the model basically the same, which can be adapted to mobile devices. Optimal results in terms of memory and computation size of the mobile device are achieved in comparison with other classical algorithms and the newly proposed lightweight transformer segmentation model. Thus, it can be seen that our newly proposed DCSANet model is well suited for field weed detection work.In **Figure 7**, we show the segmentation result plots of different models for the soybean field weed dataset. From the result plots, we can see that different models mainly differ when segmenting the boundaries of different pixels or when recognizing the overlapping of pixel categories. Both CGNet and LEDNet have the condition that the adjacent category pixels cannot be classified properly in the result plots, while MobileNetViT-Unet and VGG-Unet have the condition that the edge contours are not clear. In contrast, our proposed DCSANet can achieve both accurate detection of edge contours and reduce the cases of different types of pixels being misidentified, which indicates that our proposed model enhances the recognition accuracy of interclasses with the addition of multiscale asymmetric convolution and improves the recognition of contours at boundaries due to the enhancement of its decoding part. In summary, the DCSANet model can be well adapted to the work of weed detection in the field while keeping the model lightweight.

**Table 3:** Comparison of segmentation results of different models.

Model	*FLOPs* (*G*)	*Param* (*M*)	*MIoU* (%)
VGG-Unet	451.73	24.89	84.80
MobileNetv3-Unet	16.67	0.593	83.19
MobilenetViT-Unet	38.49	1.288	85.37
CGNet	7.11	0.492	77.66
LEDNet	12.64	2.315	82.68
DCSANet	18.06	0.57	85.95

## 4 Discussion

Many semantic segmentation algorithms have been proposed in recent years, in which algorithms with larger parameters can make good use of spatial information and make the segmentation boundaries clearer, but are not applicable to be deployed on mobile devices such as UAVs, While lightweight algorithms can meet the requirements of model deployment well, but the recognition error rate is high when weeds overlap with crops,to address this problem, we propose a novel lightweight segmentation model DCSANet,which uses an encoder-decoder structure and a DCA module with asymmetric convolution and channel shuffling on the inverted residuals structure of the MobileNetv3 model as the feature extraction backbone of the encoder part. The encoding section is divided into three layers, the goal of the first two stages is to extract feature maps containing a large amount of spatial information. To retain as much original spatial information as possible, we use only two feature extraction modules in both stages,in the third layers, we use 2 DCA-B and 6 DCA-A modules in this stage; the feature map in this stage has been downsampled several times. Therefore, the feature map has deeper feature dimensions and contains a large amount of semantic informationIn each stage of decoding, we designed a feature fusion module and borrowed the idea of guided aggregation to use high-dimensional feature maps to guide the reconstruction of low-dimensional feature maps to obtain better decoding results and to meet the accuracy requirements in field weed segmentation work, improved segmentation accuracy at target contour junctions and in areas of dense weed distribution.

We collected a soybean field weed dataset and experimentally validated our proposed DCSANet for segmentation, and the results showed that our MIou improved 2.06% over the benchmark model MobileNetv3-U-Net. The model volume was only 0.57 M, and the computational volume was only 18.06 G, which indicates that our model can readily meet the memory and computational volume requirements and achieves the best results in comparison with other classical lightweight segmentation models and recently proposed novel segmentation models, which suggests a new approach for field weed identification work. We will continue to explore how to better reduce the model size and improve the detection accuracy in the future to better contribute to further applications of smart agriculture.

## 5 Conclusions

In order to solve the problem of lightweighting the weed identification model in soybean fields so that it meets the work requirements of lightweight equipment, in this paper, we have mainly carried out the following work:

(1) A dataset of weed images from soybean fields was collected and preprocessed to simulate different real-world conditions.
(2) We propose a new lightweight segmentation model DCSAnet,the model volume was only 0.57 M, and the computational volume was only 18.06 G, which indicates that our model can readily meet the memory and computational volume requirements needs of Weed detection work.
(3) A MIoU of 85.95% was achieved on a self-collected soybean field weed dataset using DCSAnet,and achieves the best results in comparison with other classical lightweight segmentation models and recently proposed novel segmentation models,

In this paper, we have investigated the detection work of weeds in soybean fields and proposed a weed segmentation model, and in the future we will investigate the detection work of weeds in fields of other crops to increase the applicability area of the model to better contribute to further applications of smart agriculture.

## Data availability statement

The original contributions presented in the study are included in the article/supplementary materials. Further inquiries can be directed to the corresponding authors.

## Author contributions

HeY: Data curation, Supervision, Writing – review & editing. MC: Investigation, Writing – original draft. HaY: Conceptualization, Supervision, Writing – review & editing. YM: Supervision, Validation, Writing – review & editing.
